# Acute Changes in Right Ventricular Function in Pediatric Patients with Pulmonary Valve Stenosis Undergoing Percutaneous Valvuloplasty: A Speckle-Tracking Study

**DOI:** 10.3390/jcm12134344

**Published:** 2023-06-28

**Authors:** Domenico Sirico, Giulia Spigariol, Heba Talat Mahmoud, Alessia Basso, Elena Cuppini, Martina Avesani, Jolanda Sabatino, Biagio Castaldi, Giovanni Di Salvo

**Affiliations:** 1 Pediatric and Congenital Cardiology Unit, Department for Women’s and Children’s Health, University Hospital of Padova, 35128 Padua, Italy; giulia.spigariol@gmail.com (G.S.); hebatalat@ymail.com (H.T.M.); alessia.basso126@gmail.com (A.B.); elenacuppini28@gmail.com (E.C.); martiaavesani1@gmail.com (M.A.); jolesbt@hotmail.it (J.S.); b.castaldi@yahoo.it (B.C.); giovanni.disalvo@unipd.it (G.D.S.); 2 Experimental Cardiology, Paediatric Research Institute (IRP), Città della Speranza, University of Padova, 35122 Padua, Italy

**Keywords:** pulmonary valve stenosis, balloon valvuloplasty, right ventricle mechanics, longitudinal strain

## Abstract

Introduction: Pulmonary valve stenosis determines multiple effects on the right ventricular dimension and function. Percutaneous balloon valvuloplasty is the treatment of choice in severe pulmonary valve stenosis in patients of all ages. However, little is known regarding right ventricular function immediate changes after percutaneous balloon dilation. Pediatric patients with isolated pulmonary valve stenosis represent a pure clinical model of chronic RV pressure overload not affected by other confounders or comorbidities. Aim of the study: This study seeks to explore right ventricle (RV) mechanics in pediatric patients early after percutaneous balloon pulmonary valvuloplasty (BPV) for valvar pulmonary stenosis (PS). Materials and Methods: Forty-three pediatric patients (19 males), mean age 3.2 ± 4.9 years old, with severe pulmonary valve stenosis and indication for percutaneous balloon valvuloplasty were recruited. All patients underwent standard transthoracic echocardiography (TTE), and speckle-tracking echocardiography (STE) with an analysis of right ventricle free-wall longitudinal strain (RVFWLS) one day before and one day after the procedure. For each patient, we collected invasive parameters during the interventional procedure before and after BPV. Results: After the procedure, there was an immediate significant reduction in both peak-to-peak transpulmonary gradient (peak-to-peak PG) and ratio between the right ventricle and aortic systolic pressure (RV/AoP) with a drop of ∆29.3 ± 14.67 mmHg and ∆0.43 ± 0.03, respectively. Post-procedural echocardiography showed peak and mean transvalvar pressure gradient drop (∆50 ± 32.23 and ∆31 ± 17.97, respectively). The degree of pulmonary valve regurgitation was mild in 8% of patients before the procedure, affecting 29% of our patients post-BPV (*p* = 0.007). The analysis of right ventricular mechanics showed a significant improvement of fractional area change (FAC) immediately after BPV (40.11% vs. 44.42%, *p* = 0.01). On the other hand, right ventricular longitudinal systolic function parameters, TAPSE and global RVFWLS, did not improve significantly after intervention. The segmental analysis of the RVFWLS showed a significant regional increase in the myocardial deformation of the apical segments. Conclusions: Percutaneous BPV represents an efficient and safe procedure to relieve severe pulmonary valve stenosis. The analysis of the right ventricular function on echocardiography demonstrated an immediate global systolic function improvement, while longitudinal systolic function was persistently impaired 24 h after intervention, possibly due to the necessity of a longer recovery time.

## 1. Introduction

Pulmonary valve stenosis accounts for 6–9% of all congenital heart diseases. The main effect of this obstructive lesion is a rise in right ventricular pressure; this overload leads to multiple changes in the shape, dimensions, and volume of the ventricle [1,2,3,4]. The diagnosis is based on transthoracic echocardiography and invasive heart catheterization. Usually, the severity of the stenosis is based on the pressure gradient between the right ventricle and pulmonary artery and on the ratio between the right ventricle and systemic systolic pressure. Since the first description by Kan et al. in 1982 [5], percutaneous balloon valvuloplasty (PBV) has become the treatment of choice for severe pulmonary valve stenosis in patients of all ages, while surgical valvotomy remains an option in very selected cases. Furthermore, PBV has demonstrated high efficacy and safety in relieving pulmonary valve stenosis [6,7,8]. Regarding the long-term outcome after intervention, the reoccurrence of valvar stenosis has been reported to be low, while valve regurgitation after balloon dilation is more frequent [9,10,11]. Pediatric patients with isolated severe pulmonary valve stenosis constitute a pure clinical model of chronic RV pressure overload, without the influence of comorbidities. The data regarding acute post-intervention right ventricular functional changes in the pediatric population are limited [12].

## 2. Materials and Methods

### 2.1. Study Design and Population

This was a single-center study performed at the Department of Women’s and Children’s Health (W&CHD) of Padua University Hospital, Italy. The study was conducted from 2017 to 2021 and enrolled 43 consecutive pediatric patients with isolated congenital pulmonary valve stenosis and indication for percutaneous balloon pulmonary valvuloplasty (BPV) in the Cath Lab. The terms “pulmonary stenosis” and “PS” were used as keywords to search our electronic database and the collected data were subsequently validated against original medical records. The inclusion criteria were aged ≤18 years old at the time of the clinical evaluation and the presence of severe congenital pulmonary valve stenosis with indication for valvuloplasty. Patients with critical pulmonary valve stenosis (i.e., duct-dependent pulmonary circulation), patients with associated complex cardiac lesions, and patients with a previous treatment for pulmonary valve stenosis, either percutaneous or surgical, were excluded. The data of each patient were collected through institutional software Galileo (Noemalife s.p.a., Bologna, Italy), which traced the clinical data and procedural reports. Echocardiographic scans were retrieved from the institutional archive and loaded into EchoPac Software Only v204 (GE healthcare, Chicago, IL, USA) to analyze standard 2D images and for the calculation of the longitudinal strain and strain rates. Patients were then anonymized through codes, and the study data did not include personal data or identifiers. Therefore, the final dataset that was built was divided into demographic and clinical data, echocardiographic data, right ventricular strain analysis, and procedural related invasive parameters. The study was conducted in compliance with the Declaration of Helsinki principles. The local ethics committee of Padua hospital approved the study design and informed written consent was obtained from all parents or legal guardians.

### 2.2. Demographic and Clinical Data

The collected data included the baseline demographic variables (age, gender, weight, height, and body surface area) and clinical variables (non-invasive blood pressure, presence of associated cardiac lesions, and other comorbidities).

### 2.3. Standard and Speckle-Tracking Echocardiography

**Figure 1:**
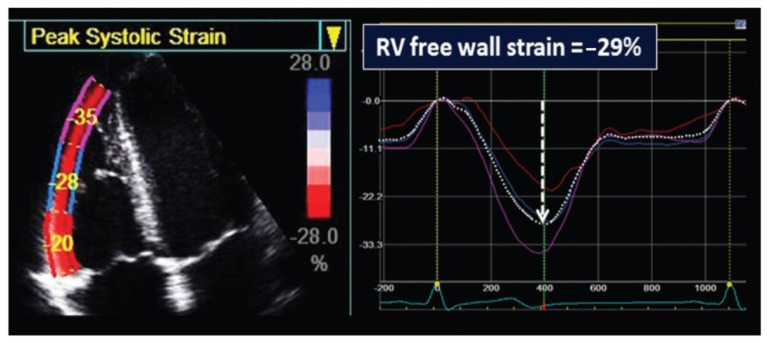
Measurement of RV free-wall systolic longitudinal strain by 2D STE. An example of right ventricle free-wall longitudinal strain measurement. In the left panel, the segmental analysis of the free-wall indicates the peak systolic negative value for each segment. The right panel shows the associated curves for each segment and the corresponding negative peak systolic average value of the longitudinal strain.

Standard two-dimensional (2D) transthoracic echocardiography was performed for all patients one day before and one day after the intervention. Scans were conducted following the present guidelines and the recommendations for cardiovascular imaging during the COVID-19 pandemic [13,14,15]. Two commercially available ultrasound machines were used: the Vivid E9 ultrasound system™ (General Electric Healthcare (GE) Wauwatosa, WI, USA) and the Philips ultrasound system (Philips Healthcare, Amsterdam, The Netherlands) with transducer’s acoustic frequencies appropriate for patient size. The right ventricle (RV) systolic function was evaluated using the tricuspid annular plane systolic excursion (TAPSE) as an estimate for the RV longitudinal systolic function and the RV fractional area change (FAC), as a measure for the RV global systolic function. The pulmonary valve was evaluated in terms of valve leaflet morphology, their mobility in 2D, and the presence of regurgitation on color-Doppler echocardiography. The annulus diameter was measured at the level of the pulmonary valve using a hinge-to-hinge measurement at the systole. The continuous-wave Doppler was used to assess the peak velocity flow across the pulmonary valve; then, the transvalvular peak and mean pressure gradients were calculated by the simplified Bernoulli’s equation. Right ventricle systolic pressure (RVSP) was also estimated from the tricuspid valve regurgitation jet peak velocity, when present, using the simplified Bernoulli equation. Moreover, RVSP was determined based on the value of the pulmonary transvalvular peak pressure gradient. Strain analysis of the right ventricle, by speckle-tracking echocardiography, was performed offline using GE EchoPac Software Only v204 (GE Healthcare, Chicago, IL, USA). Pre- and post-procedural images were analyzed offline by an expert sonographer blinded to the clinical data. Briefly, the best RV-focused 4-chamber view was used; the region of interest (ROI) was obtained by tracing the RV endocardial borders and adjusted by careful inspection of the endomyocardial boundary through performing a manual correction when necessary. Values for the right ventricular free-wall longitudinal strain (RVFWLS) by 2D STE were obtained. Longitudinal strain was defined as the percentage of systolic shortening of the myocardium from base to apex and was expressed as a negative value. The RV free wall was divided into the basal, mid, and apical segments; then, the regional peak systolic strain was automatically generated for each segment and averaged for a mean RVFWLS value (Figure 1). The strain measurements of the interventricular septum were not recorded in the present study as RV free-wall longitudinal strain alone showed a satisfying prognostic value in various diseases [16,17].

### 2.4. Interventional Procedure Data

Balloon valvuloplasty was performed, as previously reported [18]. The following hemodynamic data were collected before and immediately after BPV: right ventricle systolic pressure (RVP), main pulmonary artery systolic pressure (PAP), aortic systolic pressure or, in some cases, left ventricle systolic pressure (AoP), right ventricle to pulmonary artery (transvalvular) peak-to-peak systolic pressure gradient (peak-to-peak PG), and right ventricular to systolic systemic pressure ratio (RV/AoP). The valvuloplasty data included pulmonary valve anulus diameter on angiography, number, maximum diameter and length of the balloon catheter inflated during the procedure, and the balloon/annulus ratio. In the present study, hemodynamic immediate procedural success was defined according to the previous description as the presence of peak systolic valvar gradient reduction to <25 mmHg, a decrease in the peak pressure gradient by 50%, or a reduction in the RV/AoP pressure ratio by 50% [19].

### 2.5. Statistical Analysis

Data analyses were performed using STATA version 14 (StataCorp, College Station, TX 77845, USA). Categorical variables were expressed as number (percentage) and continuous variables were presented as mean ± SD or median (interquartile range) for skewed variables. The absolute value of strain was used to facilitate the interpretation and analysis. The chi-squared or Fisher’s exact tests were implemented to compare the categorical variables between the defined groups, as appropriate. A paired student’s *t*-test was used to determine the significance of differences in the study variables pre- and post-intervention. Linear correlation was used to compare between hemodynamic variables and 2D TTE and 2D STE. A two-sided *p*-value < 0.05 was considered statistically significant.

## 3. Results

### 3.1. Baseline Demographic and Echocardiographic Characteristics

A total of 43 patients underwent BPV during the study period with a median age and body weight of 6 months (IQR 4 days–15 years) and 7.6 Kg (IQR 2–72), respectively, of whom 19 (44%) were males. Three patients (7%) of our cohort had Noonan syndrome, 7 (16%) patients had a PFO/small ASD not hemodynamically significant, 6 (14%) had a small and restrictive patent ductus arteriosus not hemodynamically significant, and 1 had tricuspid valve dysplasia causing mild regurgitation (Table 1).

### 3.2. Procedural Data and Efficacy of BPV

The median pulmonary valve annulus diameter, as assessed by angiography, measured 9 mm (range: 6–23 mm). The median of the maximum balloon diameter used was 12 mm (range: 7–24) and the median of the maximum balloon diameter to pulmonary valve annulus ratio was 1.1 (range: 0.6–1.3). A single balloon was used in 31 (72%) patients, while 2 and 3 balloons were used in 9 (21%) and 3 (7%), respectively. Pre- and post-valvuloplasty hemodynamic data are listed and compared in Table 2. A significant decrease in the transpulmonary peak-to-peak gradient and RV/systemic pressure ratio was observed with a drop of 29.3 ± 14.67 mmHg and 0.43 ± 0.03, respectively (Table 2). Regarding the efficacy of the procedure, at least one out of the three acute procedural success criteria were present in 37 patients (86%). A reduction in the transvalvular peak-to-peak gradient to 25 mmHg or less was achieved in 74% of patients, 70% showed a peak pressure gradient reduction of 50%, and only 37% of our cohort presented a RV/AoP pressure ratio drop by 50% or more. Two out of three patients with associated Noonan syndrome had an unsuccessful procedure. After excluding these patients, the rate of success increased to 90% (36/40).

### 3.3. Echocardiographic Morphologic and Doppler Parameters

**Figure 2:**
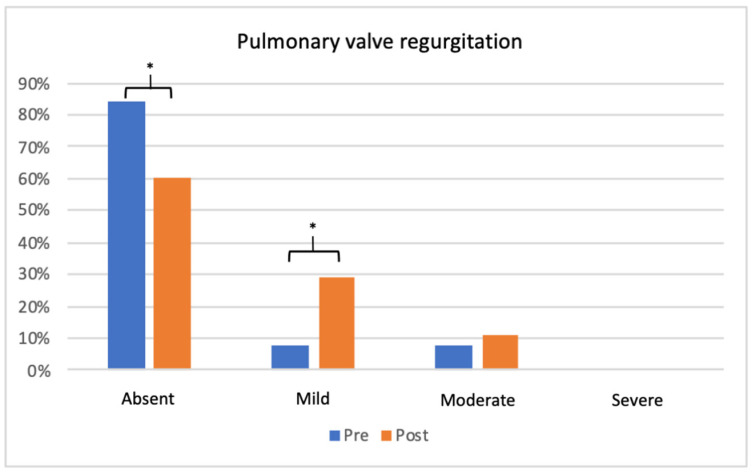
Bar graph showing the evolution of pulmonary valve regurgitation degree after balloon pulmonary valvuloplasty. Mild pulmonary regurgitation increased after the procedure, while there was no statistical increase in moderate or severe valve regurgitation before and after the procedure. * = *p* < 0.05.

Basal transthoracic 2D echocardiography showed a mean valve annulus diameter of 11.4 ± 5.6 mm, with evidence of dysplasia in 10 patients (23%). The incidence of tricuspid and pulmonary valve regurgitation before and after the procedure displayed an increase solely in the mild pulmonary regurgitation after PBV (from 8% of patients to 29% of our patients, *p* 0.007). Conversely, the incidence of moderate PV regurgitation did not worsen after the procedure (Figure 2). No studied patients showed more than a moderate PR before and after procedure. A total of 15 subjects had mild TR (35%) and there was no significant worsening of tricuspid valve insufficiency after the procedure. Right ventricle morphology (i.e., length and basal diameter) did not show significant changes after the procedure. Data obtained after BPV displayed a significant early improvement of the transvalvular pulmonary flow, in terms of peak and mean transvalvular pressure gradient drop (∆50 ± 32.2 and ∆31 ± 17.9 mmHg, respectively) (Table 3).

### 3.4. Echocardiographic Right Ventricle Functional Assessment

**Figure 3:**
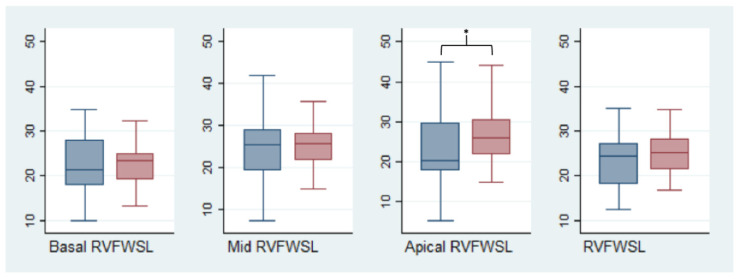
Pre- and post-procedural segmental and global right ventricle longitudinal strains. The blue box represents pre-operative values and the red boxes represent post-operative values. * = *p* < 0.05. RVFWSL = right ventricular free-wall longitudinal strain.

RV functions before and after the procedure were compared among the patients with a successful BPV procedure, as reported above (*n* = 36, 86%). Global RV systolic function measured by FAC improved after valvuloplasty (39.5 ± 7.7 vs. 44.5 ± 9.6, *p* = 0.034). No significant changes in the longitudinal RV systolic function (TAPSE) or in the RVFW longitudinal strain values were observed (13.9 ± 6.0 vs. 13.4 ± 7.2, *p* = 0.79 and −22.8 *±* 5.5 vs. −25.3 ± 6.3, *p* = 0.18, respectively). However, the segmental analysis of the RVFWLS showed a significant regional increase in the myocardial deformation of the apical segments (Figure 3, Table 4). These findings were confirmed at the subgroup analysis between the neonates/infant (*n* = 27, 63%) and children/adolescents (*n* = 16, 37%) groups, except for TAPSE, which showed a slight improvement after BPV in the younger group (10.2 ± 2.9 vs. 14.7 ± 6.1, *p* = 0.013).

### 3.5. Correlation between Hemodynamic and Echocardiographic Parameters

**Figure 4:**
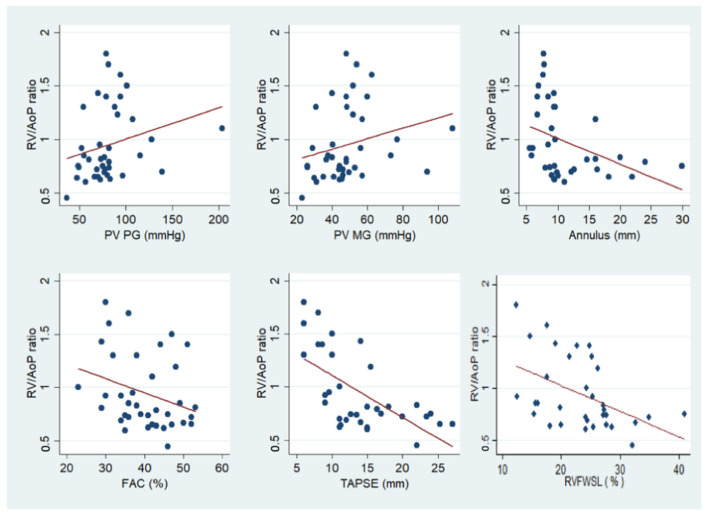
Correlations between preprocedural RV/AoP ratio and 2D TTE and STE parameters. FAC = RV fractional area change; PG = pressure gradient; PV = pulmonary valve; RV = right ventricle; RV/AoP ratio = right ventricle systolic pressure to systemic pressure ratio; RVFWSL = right ventricular free-wall longitudinal strain; TAPSE = tricuspid annular plane systolic excursion.

Univariate analysis revealed a negative correlation between the pre-operative RV/AoP ratio and pre-operative 2D TTE estimated PV annulus, and RV systolic function indices (FAC, TAPSE, and RVFW longitudinal strain). Conversely, a positive correlation was found between pulmonary transvalvular gradients (peak and mean) and the pre-operative RV/AoP ratio (Figure 4).

## 4. Discussion

The present study evaluated the functional changes in ultrasonic-derived RV systolic indexes 24 h after a successful BPV in pediatric patients affected by pulmonary valve stenosis. Our findings shed new light on the acute functional systolic changes in chronic pressure overloaded RV. The study of RV function has important prognostic implications [20]; thus, a better understanding of its adaptation to chronic pressure overload and acute unloading may be of clinical value for many clinical conditions. Nevertheless, RV functional quantification is challenging due to RV complex anatomy and structure. Speckle-tracking imaging is a well-established echocardiographic tool able to assess myocardial wall deformation by strain; it is less affected by geometrical and loading confounders [21]. We found that the acute pressure unloading of the right ventricle after BPV determined a significant early improvement in global RV function measured by the FAC, while longitudinal RV systolic function, as assessed by TAPSE and RVFWLS, did not show a significant early change. These findings may be explained by the load dependence of FAC, while TAPSE and RVFWLS were less loading-dependent. However, our data may also suggest that RV longitudinal fibers are more sensitive to pressure overload; conversely, the increase in FAC, an index expressing the combination of longitudinal and radial functions, may also suggest an earlier recovery of circumferentially oriented RV fibers. In agreement with our findings, a recent study on 15 infants undergoing BPV for severe PS, by Gozar et al., did not show a significant difference in the RV free-wall longitudinal strain before and 24 h after the procedure [12]. Regarding the segmental analysis of the RV longitudinal strain, in healthy subjects, it was described as a basal-to-apex gradient, the basal segments being more negative [22,23]. In our cohort, we did not find a statistically significant gradient before the procedure. On the other hand, we measured a significant increase in the regional strain of RVFW apical segments early after the procedure, compared to mid and basal ones. These data suggest that RVFW apical segments’ longitudinal strain might show an earlier improvement compared to basal and mid ones after pulmonary stenosis relief. This finding may be explained by the lower parietal stress acting on the apical segments due to the short RV diameter at the apical level. Our findings might be of interest in the early treatment evaluations of chronic RV pressure overload conditions (e.g., pre- and post-capillary pulmonary artery hypertension, chronic pulmonary embolism, etc.), giving the clinician an adjunctive indicator of therapeutic benefits, either medical or interventional, and efficacy. In this regard, cardiac MRI has demonstrated to be a reliable tool for the assessment of RV global longitudinal and radial strains in a population of patients with repaired Tetralogy of Fallot and may overcome some of the limitations of transthoracic echocardiography [24]. Moreover, it is reported that long-standing RV pressure overload may lead to myocardial fibrosis assessed on cardiac MRI, and this finding can possibly jeopardize the benefits of pressure overload relief [25]. However, in our cohort, we did not find significant differences in terms of RV functional recovery after BPV between younger and older children. Finally, the present analysis demonstrated a significant correlation of the pre-procedural invasive right ventricle to systemic pressure ratio with numerous echocardiographic parameters, including valve annulus, peak and mean systolic gradients, and global and longitudinal RV systolic function indices (i.e., FAC, TAPSE). Moreover, we found that RVFW longitudinal strain is significantly correlated with the RV/AoP pressure ratio, as previously described by Kawakubo et al. [26]. Therefore, the RV/Ao pressure ratio should be used together with the peak-to-peak transvalvular invasive pressure gradient (ΔP) for a thorough invasive evaluation of pulmonary valve stenosis severity.

### Study Limitations

Our sample size may seem small; however, to study the changes induced by BPV in a pure clinical model of RV chronic pressure overload, we only selected pediatric patients. We excluded patients with associated congenital abnormalities (other than hemodynamically insignificant PFO and PDA), and we only included patients for whom BPV was successful. In addition, all the echo measurements were conducted 24 h before and 24 h after the procedure in the whole studied sample. Sample age heterogeneity may constitute a limitation, as it ranged from 4 days to 15 years. The timing of the post-procedural ultrasound evaluation may represent a limitation, and further studies need to investigate our findings at the long-term follow-up.

## 5. Conclusions

Our study, in a pure clinical model of chronic RV pressure overload exposed to successful acute pressure unloading, suggests that longitudinal function impairments persist longer than 24 h and the functional recovery starts from the RVFW apical segments.

## Figures and Tables

**Table 1:** Demographic data.

Demographic Data	Value *
Age (years)	0.5 (0.1–4.0)
Age groups	
Neonates (%)	9 (21)
Infants (%)	18 (42)
Children (%)	11 (25)
Adolescents (%)	5 (12)
Female gender (%)	24 (56)
Weight (kg)	7.6 (4.8–15.7)
BSA (m^2^)	0.2 (0.1–0.6)
Comorbidities	
Noonan syndrome	3 (7)
PFO/small ASD	7 (16)
Small PDA	6 (14)
Tricuspid dysplasia	1 (2)

* Data reported as frequency (%) and median (interquartile range). BSA = body surface area calculator. ASD = atrial septal defect. PDA = patent ductus arteriosus. PFO = patent foramen ovale.

**Table 2:** Pre- and post-procedural hemodynamic data.

	Pre Valvuloplasty	Post Valvuloplasty	*p*-Value
Peak-to-peak PG (mmHg)	49.1 ± 13.7	18.3 ± 10.7	<0.001
RV/AoP ratio	1.0 ± 0.4	0.5 ± 0.2	<0.001

Data reported as mean ± SD. PG = pressure gradient; RV/AoP = right ventricular to systemic pressure ratio.

**Table 3:** Echocardiographic morphologic and Doppler parameters.

2D TTE	Pre-Procedure	Post-Procedure	*p*-Value
Pulmonary regurgitation			
Mild (%)	3(8)	11 (29)	0.007
Moderate (%)	3(8)	4 (11)	0.15
Severe (%)	0 (0)	0 (0)	1
PV peak velocity (m/s)	4.4 ± 0.8	2.9 ± 0.7	<0.001
PV Peak PG (mmHg)	81.1 ± 29.5	35.5 ± 17.3	<0.001
PV Mean PG (mmHg)	48.2 ± 17.3	20.1 ± 9.9	<0.001
RVSP (mmHg)	83.0 ± 30.8	37.3 ± 17.0	<0.001
RV length (mm)	38.7 ± 13.7	38.7 ± 13.6	0.59
RV basal diameter (mm)	22.6 ± 8.0	22.3 ± 7.7	0.97

Data reported as frequency (%) and mean +/− SD. 2D TTE = two-dimensional transthoracic echocardiography; PG = pressure gradient; PV = pulmonary valve; RV = right ventricle; RVSP = right ventricle systolic pressure.

**Table 4:** Right ventricle TTE and STE functional assessments in patients with successful procedure.

2D TTE	Pre-Procedure	Post-Procedure	*p*-Value
FAC (%)	39.5 ± 7.7	44.5 ± 9.6	0.034
TAPSE (mm)	13.9 ± 6.0	13.4 ± 7.2	0.79
**2D STE**			
RVFWSL (%)	−22.8 ± 5.5	−25.3 ± 6.3	0.18
Basal (%)	−23.0 ± 6.5	−23.1 ± 6.2	0.95
Mid (%)	−24.0 ± 6.7	−26.3 ± 8.0	0.33
Apical (%)	−21.4 ± 7.5	−26.6 ± 7.5	0.03

Data reported as frequency (%) and mean +/− SD. 2D STE = two-dimensional speckle-tracking echocardiography; 2D TTE = two-dimensional transthoracic echocardiography; FAC = RV fractional area change; RVFWSL = right ventricular free-wall longitudinal strain; TAPSE = tricuspid annular plane systolic excursion.

## Data Availability

The data presented in this study are available on request from the corresponding author. The data are not publicly available.
